# Media and education play a tremendous role in mounting AIDS awareness among married couples in Bangladesh

**DOI:** 10.1186/1742-6405-4-10

**Published:** 2007-05-12

**Authors:** Mohammad Shafiqur Rahman, Mohammad Lutfor Rahman

**Affiliations:** 1 Institute of Statistical Research & Training, University of Dhaka, Dhaka-1000, Bangladesh

## Abstract

**Background:**

To quarantine the spreading possibility of HIV virus to general population boosting public awareness is must. But the proper awareness level is substantially low in Bangladesh. This paper aims to identify the factors associated with the awareness regarding HIV/AIDS through a bivariate and multivariate analysis using the data extracted from Bangladesh Demography and Health Survey (BDHS) 1999–2000.

**Results:**

The findings of both techniques show that education, occupation, socioeconomic status, status of household food consumption, area of residence and media exposure have significant (p < 0.001) contribution in determining HIV/AIDS awareness level. It also reveals that media, particularly TV, and education play the leading role regarding this issue while the others have an indirect relationship. The odds of awareness among higher educated women and men were 4.69 and 77.73 times of no educated women and men respectively. In addition, both women and men those who regularly watch TV were 8.6 times more likely to be aware about AIDS compared to those who never watch TV. This phenomenon holds true for both women and men.

**Conclusion:**

At this instant it is urgent to give emphasis on education, alleviation of poverty, ensuring electronic media exposure, head to head communication program, institutional based sex education and necessary information to learn about HIV/AIDS for the young, adult and adolescents all over the country.

## Background

Acquired immunodeficiency syndrome (AIDS) is an infectious disease caused by human immunodeficiency virus (HIV) has created a major global health crisis and its impact on a country is tremendous [1-3]. The world has already experienced the overwhelming downbeat impact of HIV/AIDS epidemic on the development of severely affected countries [2,3]. Bangladesh is still considered as a low HIV/AIDS prevalent country but it is at a critical moment in the course of its AIDS epidemic [1,4-8]. According to UNAIDS estimates, Bangladesh, with a population of 136 million, had about 13,000 people living with HIV/AIDS at the end of 2001 and that HIV prevalence in the adult population is less than 0.01% [9]. Although overall HIV prevalence is low, Bangladesh is considered a high-risk country for several reasons: the presence of covert multi-partner sexual activity and denial, the low level of knowledge and low condom use, unsafe professional blood donation, high incidence of self-reported sexually transmitted infections among vulnerable groups, coming back of expatriates working in different countries, and high levels of HIV/AIDS in the two neighbouring countries, India and Myanmar, all contribute to the spread of HIV [4-8,10]. On the other hand, the country's vulnerability is very high compared to other parts of South Asia and infection rates within the vulnerable groups are increasing, leading to an ever-greater possibility that the virus will spread to the general population [4,5,11]. In this critical situation, public awareness can play a dominating role preventing HIV/AIDS epidemic [12]. But awareness level with knowledge of correct ways to avoid HIV/AIDS among the general people in Bangladesh is quite low. Among the men with age 15–54, 18% never heard HIV/AIDS, 24% ever heard but don't know any correct ways to avoid it and only 58% knows one or more correct ways to avoid the disease [13]. On the other hand, 40% ever-married women never heard HIV/AIDS, 19% ever heard but don't know any correct ways and only 41% knows one or more correct ways to avoid the disease [13]. These situations have raised serious concern among the government and various stakeholders and they are seeking to increase the public awareness on HIV transmission and prevention. So, it is important to identify the reasons that associated with level of awareness, which will be helpful in strengthening Govt./NGO/development-partner agencies' capacity for program planning, implementation, monitoring and evaluation regarding AIDS awareness. In this regards a few national and international researchers have made attempts to understand the reasons and come up with some explanations [12,17,18]. But existing analysis didn't identify noticeably for which factors AIDS awareness modifies significantly in context of Bangladesh. It is with the background, the present study has design to examine the factors that are associated with level of awareness regarding AIDS prevention.

## Data and methods

The data for this study were obtained from the 1999–2000 Bangladesh Demographic and Health Survey (BDHS). It is a cross-sectional survey that have been carried out once in every two years since 1993 among a nationally representative samples of women as done in many other countries. The BDHS is part of the worldwide Demographic and Health Surveys (DHS) programme which collects information on a number of areas such as demographic characteristics, reproductive history and family planning. The survey was conducted during the period from November 1999 to March 2000, under the authority of the National Institute of Population Research and Training (NIPORT), Bangladesh. A nationally representative two-stage probability sample design was used for the sample survey in which a total of 10544 currently married women and 2556 currently married men were successfully interviewed. Details of the methodologies adopted in BDHS can be found elsewhere [14]. This study is based on 10544 currently married women, who are 10–49 years old and 2556 currently married men.

Since a cure or vaccine is unlikely in the near future, efforts to prevent the HIV epidemic must focus on public awareness. Several countries, including Thailand and Uganda, have successfully decreased the spread of HIV by aggressive efforts in this regard [15]. Keeping the above in mind, the present study has aimed to examine the association between AIDS awareness and a set of independent variables. The set of independent variables are women educational attainment, current engagement in an income generation activity, husband education, occupation, household food consumption, number of household assets (e.g, radio, TV, bicycle etc.) which determine the socioeconomic status of the household, along with some programmatic variables such as number of visits by family planning field worker (FPFW), number of visit by health field worker (HFW) in the last six months preceding the survey and how often listen radio, how often watch TV. The dependent variable, 'ever heard AIDS' used to determined the status of HIV/AIDS awareness, was coded as 1 for yes as 0 for not at all. Separate analysis has been carried out for male and female respondents for existence of different background information of male and female respondents. Both bivariate and multivariate techniques have been performed to assess the factors associated with AIDS awareness in Bangladesh. Chi-sqaure test is used to determine the association between dependent and independents variable as bivariate techniques. Logistic regression [16] was carried out as multivariate techniques to assess the net effects of independent variables on AIDS awareness level. In the logistic regression analysis all the independent variables are coded as categorical variables and dummy coding scheme was also used. Odds ratio has been used to compare different groups.

## Results

Appendix Table 1 shows the distribution of both males and females who ever heard HIV/AIDS by various independent variables. The corresponding results of logistic regression analysis are presented in Table 2 and Table 3.

**Table 1:** Distribution of respondents who ever heard AIDS by various independent variables

	**Female**		**Male**		
**Variable**	**No of females**	**% of females who heard AIDS**	**Variable**	**No of males**	**% of males who heard AIDS**

Education*			Education*		
No education	4842	12.4	No education	891	20.4
Primary	1928	23.2	Primary	782	25.6
Secondary	1074	35.7	Secondary	590	39.2
Higher	2699	72.0	Higher	293	82.1
**Currently working**			Respondent Occupation*		
Yes	8164	31.3	Didn't work	56	55.4
No	2377	28.9	Prof., tech., mang.	706	72.0
			Agric-self employed	754	36.5
			Agric-employee+manual work	1023	30.6
Husband education*			HH items*		
No education	4181	12.9	0–3	854	8.5
Primary incomplete	1471	20.3	4–6	981	29.2
Primary complete	923	28.5	7–12	811	68.5
Secondary+	3952	54.1			
Husband Occupation*			HH food consumption*		
Didn't work	652	45.4	Deficit in whole yr	450	16.5
Prof., tech., mang.	2933	49.1	Sometimes deficit	720	26.4
Agric-self employed	2509	14.7	Surplus or equal	1386	65.8
Agric-employee +manual work	4449	25.6			
HH items*			How often listen radio*		
0–3	3399	10.5	Never	1065	37.5
4–6	3571	24.2	Sometimes	837	53.6
7–12	3573	56.6	Every day	651	67.0
HH food consumption*			How often watch TV*		
Deficit in whole yr	1735	14.5	Never	954	26.5
Sometimes deficit	4339	22.4	Sometimes	1029	54.0
Surplus or equal	4470	45.3	Every day	571	83.2
How often listen radio*			Area of residence*		
Never	6821	21.0	Urban	508	76.4
Sometimes	1962	45.6	Rural	2048	43.8
Every day	1759	52.1			
How often watch TV*			NA		
Never	6164	12.5			
Sometimes	2327	38.5			
Every day	2050	77.1			
**No. of visits by FPFW**			NA		
0	8465	30.8			
1	482	30.3			
2+	1596	31.3			
**No. of visits by HFW**			NA		
0	8963	31.0			
1	486	31.3			
2+	1094	29.3			
Area of residence*			NA		
Urban	2070	64.3			
Rural	8473	22.6			

*p < 0.001

**Table 2:** Logistic regression results of AIDS awareness for female respondents

				**95% CI for odds ratio**	
				
**Variable**	**Coefficient**	**P-value**	**Odds ratio**	**Lower**	**Upper**
**Education**		.000			
No Education			1.000		
Primary	.361	.000	1.435	1.233	1.671
Secondary	.621	.000	1.861	1.561	2.219
Higher	1.543	.000	4.679	4.021	5.444
**Current working status**		.000			
No			1.000		
Yes	.227	.001	1.255	1.103	1.428
**Husband Education**		.000			
No Education			1.000		
Primary	.181	.042	1.199	1.007	1.428
Secondary	.376	.000	1.457	1.200	1.770
Higher	.614	.000	1.848	1.595	2.142
**Husband occupation**		.000			
Didn't Work	1.116	.000	3.051	2.424	3.841
Prof., Tech., Mang.	1.098	.000	2.999	2.570	3.501
Agric-self employed			1.000		
Agric- employee+manual work	.922	.000	2.514	2.160	2.927
**HH items**		.000			
0–3			1.000		
4–6	.403	.000	1.496	1.278	1.752
7+	1.023	.000	2.780	2.338	3.306
**HH food consumption**		.015			
Deficit in whole year			1.000		
Sometimes deficit	.061	.506	1.063	.888	1.271
Surplus or equal	.214	.024	1.239	1.029	1.492
**How often radio**		.000			
Never			1.000		
Sometimes	.408	.000	1.504	1.319	1.714
Everyday	.637	.000	1.891	1.650	2.167
**How often TV**		.000			
Never		.993	1.000		
Sometimes	1.059	.000	2.883	2.540	3.272
Everyday	2.149	.000	8.576	7.386	9.957
**No. of time visited by FP FW**					
0			1.000		
1	-.008	.936	.992	.815	1.208
2+	-.035	.822	.966	.712	1.309
**No. of time visited by Health FW**		.646			
0			1.000		
1	-.026	.837	.975	.762	1.246
2+	.068	.694	1.070	.763	1.501
**Area of residence**					
Urban	1.367	.000	3.925	3.442	4.447
Rural			1.000		
Constant	-3.913	.000			

**Table 3:** Logistic regression results of AIDS awareness for male respondents

				**95% CI for odds ratio**	
				
**Variable**	**Coefficient**	**P-value**	**Odds ratio**	**Lower**	**Upper**
**Education**		.000			
No Education			1.000		
Primary	.724	.000	2.063	1.639	2.596
Secondary	2.200	.000	9.029	6.857	11.889
Higher	4.353	.000	77.733	31.690	190.668
**Resp. occupation**		.000			
Didn't Work	.295	.361	1.343	.713	2.530
Prof., Tech., Mang.	.670	.000	1.954	1.477	2.585
Agric-self employed			1.000		
Agric- employee+manual work	.314	.011	1.368	1.073	1.744
**HH items**		.000			
0–3			1.000		
4–6	1.230	.000	3.278	1.278	5.752
7+	2.275	.000	8.521	3.028	11.425
**HH food consumption**		.005			
Deficit in whole year			1.000		
Sometimes deficit	.061	.506	2.063	.888	1.271
Surplus or equal	.214	.024	5.239	1.029	1.492
**How often radio**		.000			
Never			1.000		
Sometimes	.381	.002	1.464	1.155	1.856
Everyday	.679	.000	1.972	1.516	2.566
**How often TV**		.000			
Never		.993	1.000		
Sometimes	1.059	.000	3.253	2.540	3.272
Everyday	2.148	.000	8.582	7.386	9.957
**Area of residence**					
Urban	.970	.000	2.65	1.99	3.53
Rural			1.000		
Constant	-2.857	.000			

### Female

Bivariate results for female respondents showed that other than woman's current working status, number of visit by family planning field worker (FPFW) and health field worker (HFW), all the other variables included in this analysis had statistically significant relationship with the level of HIV/AIDS awareness (Table 1). Nevertheless, the logistic regression results identified education, partner's occupation, household economic status as measured by assets owned, status of household food consumption, electronic media like radio and TV and area of residence as having statistically significant relationship with AIDS awareness (Table 2).

Awareness regarding HIV/AIDS was highest among higher educated women and lowest among the illiterate women. Women with no education only 12.4% ever heard AIDS. As the education level increases the percent of the respondents who aware about AIDS also increases. Women with education secondary or more belongs to highest percent, 72.0%, of awareness. Often awareness of the women relies on their husbands' education and occupation. The findings shows that the wives of the husbands with education secondary plus are more conscious, 54.1%, than those having the husbands with no education, 12.9%. In relative sense the odds of awareness among the women with education level higher were 4.68 times of the women with no education. In addition, wives of the educated husbands know better about AIDS than the wives of the uneducated husbands and this difference is about 85% more in the literate group.

Wives of the husbands employed in agriculture and/or does manual work bear least knowledge as to AIDS than wives of the husbands employed as professional (doctor, teacher, banker etc), technical expert and managerial work. Among the wives of the agricultural self-employees only 14.7% learnt about AIDS, followed by agricultural employee or manual work, 25.6%. The percentage is high in the category like professional/technical/managerial, 49.1% and didn't work, 45.4%.

Household economic status determined by assets owned has a positive affect on awareness. Women belonging to the households with 7–12 items have the awareness more, 56.6% and less in the group having 0–3 items, 14.5%. In the comparative sense the women of the households with 7 or more items were 2.8 times more alert than the households with 3 or less items.

Women of the households with food surplus or equal are more conscious, 45.3%, than the women of the households with food deficit sometimes, 22.4% or deficit in the whole year, 14.5%. In addition, the odds of awareness was 1.2 times among women of the household with food surplus or equal compare to those women of the household with food deficit in the whole year.

It is usual to have awareness among the people who retain themselves in close contact to mass media/electronic media like radio, TV. Women who listen to radio everyday possess the highest percent, 52.1%, in awareness and 45.6% who listen often and 21.0% who listen never. Of everyday female TV watchers 77.1% were alert about AIDS, 38.5% of those who watch TV sometimes and only 21.0% of those who never do so. In comparative sense, the odds of awareness were 1.9 and 1.5 times among the women who had listen radio every day and listen sometimes respectively compare to those who had never listen radio. Correspondingly the odds of awareness among regular TV viewers and moderate viewers were 8.6 and 2.9 times respectively of those never watch TV.

It is usual to expect that urban people are more alert than their rural counterparts. Urban people enjoy more amenities like TV, telephone, radio, newspaper, magazine etc. than rural people. They are closer to the information highway than the rustics. Women living in the urban areas have more awareness, 64.3%, than those who live in the rural areas, 22.6%. In relative sense, urban women were 3.93 times more aware compare to its rural counterpart.

### Male

Bivariate results for male respondents on the other hand showed statistically significant relationship between education, occupation, household economic status as measured by assets owned, household food consumption, media like radio and TV, area of residence and AIDS awareness (Table 1). Logistic regression results for male respondents picked up all variables included in the analysis as statistically significant variables (Table 3). The results found here are almost similar with the result of female respondents.

Educated males also have more awareness in comparison to males with no education. Among males with education higher secondary or more, 82% heard about AIDS. The figures are 39%, 25% and 20% for the males with secondary, primary and no education respectively. In comparative sense, the odds of awareness among the men with higher education, secondary education and primary education were 77.7, 9.0 and 2.1 times respectively of those who have no education.

Occupation determines largely the social status of an individual. Awareness level varies with their professions. Of the male respondents with professions like teaching, managing and technical 72% heard about AIDS and the percentage is less in agricultural employees and manual workers, 30.6%. On the other hand, Professionals like teachers, engineers, managers etc. are 1.95 times more aware than agricultural self employed males.

On the other hand, household possessions reflect the socioeconomic index of a household, which relates the consciousness as well as AIDS awareness. The more the household items the more the percentage of people who have heard AIDS. In addition, the odds of consciousness among the men of household with 7 or more items was 8.5 times of those men who belonging the household with three or less items while the odds was 3.2 times among men whose household contained 4–6 items. The awareness as to HIV/AIDS also indirectly related to food consumption pattern. It is found that households with food deficiency in the whole year have one-fifth awareness in comparison to the households with food surplus or break-even point as well as households with food deficiency hardly or off and on.

Media like radio and TV also have a strong positive relationship with awareness. Everyday male listeners of radio have highest-level awareness, 67.0%, followed by the moderate listeners compared to 37.5%, who had never listen radio. In addition, the odds of awareness among regular listeners and moderate listeners were 2.0 and 1.5 times of those who had never listen radio.

TV watching have more direct impact on awareness of male. Among everyday watchers 83.2% are concerned of AIDS. This figure is far away than those who did never, 26.5%. In relative sense, the odds of consciousness were 8.6 and 2.9 times among the men who watch TV regularly and watch sometimes respectively compared to those men who never watch TV. Men living in the urban area were also more aware than its rural counterpart.

## Discussion

From both bivariate and multivariate techniques, the study has identified the factors exposure of electronic media, education of the respondent, partner's education, area of residence, occupation, socioeconomic status determined by household possessions, and food consumption pattern that were associated with the level of awareness about HIV/AIDS. This phenomenon holds true for both male and female respondents.

**Figure 1:**
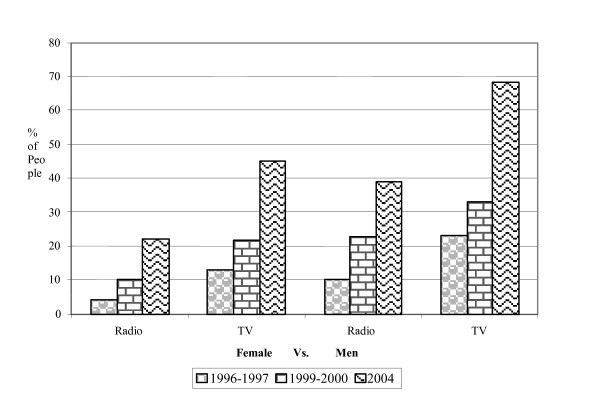
Increasing trend in proportion of peoples identified media (Radio and TV) as main source of AIDS information.

Broadcast media like radio, TV have tremendous reach and influence and play a vital role to build up awareness against HIV/AIDS in the community [17,18]. According to BDHS reports 1996–2004, there is an increasing trend in proportion of women identified media (both radio and TV) as their main source of information about AIDS (Figure 1). It has seen that 68% men and 45% women in 2004 identified television as their primary source of information about HIV/AIDS compared to 22% and 12% respectively in 1996. Similar scenario has also been observed for radio (Figure 1).

Analogous statistics have also been reported in the United States and United Kingdom and elsewhere in the world [17,19]. This indicates that media organizations have an enormous influence in educating and empowering individuals to avoid contracting HIV/AIDS. This study also reveals that the electronic media like radio and TV, particularly TV, play leading role in building awareness about AIDS. But only 31.6% households in Bangladesh have a radio while 17.5% have a TV [14]. Again only 35% women and 53% men watch television while 29% women and 53% men listen to the radio at least once a week [14]. Thus, low percent of radio/TV owner and also low percent of listener/viewer indicates to have low awareness. Hence, media exposure should be maximized to increase awareness. Then it should be promoted the people to listening/watching health issued programs on radio or TV regularly.

Education is an event of human life that carries out a significant role in determining his/her social status. In the context of Bangladesh, high education indicates better occupation, better income and better income eases media access. More exposure in media signifies more awareness about HIV. Again socioeconomic status determined by existent household assets including radio/TV and household food consumption, also has a contribution to determine HIV/AIDS awareness level. It is found from the study that poor people, determine by household's food deficiency in the whole year and/or number of household items, are less likely to be aware of AIDS as likely as its counter rich peoples. It is due to the facts that poor people have less education and less media exposure than rich people. Thus, poverty alleviation would be another strategy to increase awareness.

In Bangladesh urban areas are much more developed than rural areas in terms of socioeconomic factors like education, occupation, income, media exposure, health service facility etc. Findings of the study show that both urban male and female are much more aware about HIV/AIDS than rural counterparts. Therefore, more socioeconomic development indicates more awareness. But in context of developing country like Bangladesh, the rural peoples have less media contact. Since about 76% people live in rural area with less media coverage, it should give special attention to develop socioeconomic condition of rural people, which may meet people to media, and as a result there will be a remarkable change in awareness level. In contrast, as the use of mass media such as radio, TV is very limited in Bangladesh especially in rural areas as compared to urban areas, some additional programs such as face-to-face communication and sexual education at institutions may be effective in raising awareness in Bangladesh.

## Conclusion

Therefore, now is the time to emphasize more on education, alleviation of poverty, ensuring electronic media exposure, head to head communication program, institutional based sex education and necessary information to learn about HIV/AIDS for the young, adult and adolescents all over the country. On the other hand, for effective use of mass media, it requires careful planning, audience research, message development, pre-testing, dissemination strategy, evaluation, co-ordination with existing services, and linking mass media with interpersonal communication. The role of international health and development organizations in promoting, supporting and advocating the use of well-planned mass media campaigns can also make a significant difference [18]. All possible venues such as workplace, schools, mosques/churches/temples, etc should be targeted to intensify health promotion and education activities. Social and religious values and attitudes should be maximized for creating more supportive environments for HIV/AIDS prevention. As HIV/AIDS comes and kills us silently and any one can be infected any time by this tremendous enemy in absence of proper awareness, every cautious and alert person needs to participate as an active soldier in the battle of HIV/AIDS prevention through massive awareness building in Bangladesh.
